# Short Communications: Automated dissolution rate analysis of iron in some vitamin preparations

**DOI:** 10.1155/S1463924679000432

**Published:** 1979

**Authors:** Bo Karlberg, Sidsel Thelander

**Affiliations:** Astra Pharmaceuticals Analytical Control Sodertalje S-151 85 Sweden


Since the end of the evaluation the assay has been in routine use and no problem has been experienced, although the reagents were from the same batch. Further clarification of the difficulty is required.

Original studies on the uric acid methodology were performed using the Boehringer Urica-quant kit, as recommended by Coulter, but the correlation studies showed poor agreement with standard techniques due primarily to a non-linear reaction course. This phenomenon had been reported previously in an evaluation study on the Abbot ABA-100 analyser by Bullock *et al* [17]. It was for this reason that a further study on uric acid measurement was performed using an NADP coupled reaction as detailed by SKI. This is a two-reagent method, uricase being the starter reagent, and is based on the oxidation of acetaldehyde by aldehyde dehydrogenase in the presence of NADP. On the Kem-O-Mat the reaction was found to be linear to 1500 $\mu$mol/l showing a good correlation with the routine method.

The creatine kinase assay would appear to give poor between-batch precision, but of the same order as that obtained in the routine laboratory; the difficulty is a reflection of the chemistry and not the functioning of the instruments involved.

The reagent costs given in Table 8, show the best and worst cases of reagent utilisation. The manufacturer quotes figures for total reagent consumption whereas the figures found in use are determined from opening a single vial of reagent; the true reagent cost probably falls somewhere between the two. Some of these costs would appear to be high, but when compared with the costs on other instruments in the author's laboratory a net saving can be made on selected chemistries in which expensive reagents are used.

The Kem-O-Mat was found to give an acceptable performance both during the evaluation period and in subsequent use.

ACKNOWLEDGEMENTS
Thanks are due to: Coulter Electronics Ltd., for the loan of the instrument, the supply of reagents and consumables, and help during the evaluation and preparation of this report; Mr. R.J. Wood for his work in testing the electrical and mechanical safety of the instrument as well as the construction of the thermistor assembly; Mr. B. Hawgood for the supply of the microbial suspension, settle plates and subsequent interpretation of the relevant results; to Dr. M.G. Rinsler, Dr. S.S. Brown and Dr. F.L. Mitchell for encouragement and critical comment of the report.

REFERENCES

[1] Broughton, P.M.G., Buttolph, M.A., Gowenlock, A.H., Neill, D.W. and Skentelbery, R.G., *Journal of Clinical Pathology,* 1969, 22, 278.
[2] Broughton, P.M.G., Gowenlock, A.H., McCormack, J.J. and Neill, D.W., *Annals of Clinical Biochemistry,* 1974, **11**, 207.
[3] Trinder, P., *Journal of Clinical Pathology,* 1969, 22,246.
[4] Bowers, G.N. and McComb, R.B. *Clinical Chemistry,* 1966, **12**, 70.
[5] Allain, C.C., Poon, L.S., Chan, C.S.G., Richmond, W. and Fu, P.C., *Clinical Chemistry,* 1974, **20**, 470.
[6] Bergmeyer, H.U. *Journal of Clinical Chemistry and Clinical Biochemistry, 1975,* **13**, 507.
[7] Peterson, J.I. and Young, D.S., *Analytical Biochemistry,* 1968, 23,301.
[8] Bucolo, G. and David, H., *Clinical Chemistry,* 1973, **19**, 476.
[9] Kageyama, N., *Clinica Chimica Acta,* 1971,**31**, 421.
[10] Haeckel, R.J., *Journal of Clinical Chemistry and Clinical Biochemistry,* 1976,**14**,101.
[11] Kind, P.R.N. and King, E.J., *Journal of Clinical Pathology,* 1954,7, 322.
[12] Seymour, G.C. and Gray, C.J., *Medical Laboratory Sciences,* 1978, **35**,55.
[13] Wahlefeld,, A.W., in 'Methods of Enzymatic Analysis' Ed. Bergmeyer, H.U., 1974, Adademic Press p. 1831.
[14] Morin L.G., *Clinical Chemistry,* 1974, **20**,51.
[15] Richmond, W., *Clinical Chemistry,* 1976, **11**,1579.
[16] 'Electrical Safety Code for Hospital Laboratory Equipment.' 1977, H.M.S.O., London.
[17] Bullock, D.G., Badham, L.P. and Wilding, P., 'An evaluation of the Abbott Biochromatic Analyser'. Special Report No. 2. Wolfson Research Laboratories, Queen Elizabeth Medical Centre, Birmingham, England.
[18] Bowers, G.N., Jr., Bergmeyer, H.U., and Moss, D.W., *Clinica Chimica Acta, 1975,* **61**,F11.

### Short Communications

### Automated dissolution rate analysis of iron in some vitamin preparations

**Bo Karlberg and Sidsel Thelander**

Astra Pharmaceuticals, Analytical Control, S-151 85 Sodertalje, Sweden.

Dissolution rate analysis (DRA) is often required in the quality control of slow-release preparations. Manually this type of analysis is very tedious and time-consuming. Shah, et al [1], have summarized the requirements that are necessary on a system for DRA. For instance, the withdrawal of the samples must be made without interrupting the agitation of the solution. In many systems sample acquisition is performed continuously. The test solution is pumped through a filter into a flow cell and then back to the dissolution vessel. This is a convenient arrangement for some applications but it has a severe limitation. If the active constituent does not possess absorption properties within UV or visible regions an additional treatment of the sample is necessary. In this case the sample will be destroyed. The aliquots must therefore be removed at known times and analysed later. Furthermore, they must be so small as to avoid a significant change in the volume of the dissolution fluid.

This paper describes a fully automated system for DRA of iron in some vitamin preparations. The dissolution is determined at one, two and four hours. The system is commercially available* and very versatile since the analytical cartridge can be modified easily. For example, potassium in slow release preparations can be determined by using a flame photometer detector [2].

### Materials and method

### Reagents

All chemicals should be of Reagent grade.

*R 1: Hydroxylamine in 2 M hydrochloric acid.*
Weigh 12.5 g hydroxylamine into a 250 ml volumetric flask. Make up volume with 2 M hydrochloric acid. This amount of solution is sufficient for two complete runs.

*R 2: Buffer at pH 5.0*
Weigh 272.2 g sodium acetate into a 1 litre volumetric flask. Make up volume with water. Add 2 M acetic acid to this solution until pH 5.0 is reached. Add 1 ml of wetting agent (Brij 35) per litre of the buffer. Discard the solution at first sign of turbidity.

*Technicon Instruments Inc.*

*R 3: 0.1% 1-10-phenanthrolinium chloride*

Weigh 0.25 g 1-10 phenanthrolinium chloride monohydrate into a 250 ml volumetric flask. Dissolve in water. Add 0.25 ml of wetting agent (Brij 35) and make up volume with water. Discard the solution at first sign of purple colour.

### Standard solutions

The tablet specification is 100 mg iron per tablet. The dissolution is expressed as a percentage of this specification. It is therefore advantageous to make standards accordingly.

*Standard, 25%:* Weigh 1.7553 g ammonium iron (II) sulphate into a 1 litre volumetric flask. Add 20 ml 2 M hydrochloric acid. Fill up with water.

*Standard, 50%:* As above, but with 3.5107 g ammonium iron (II) sulphate.

*Standard, 75%:* As above, but with 5.2660 g ammonium iron (II) sulphate.

### Equipment

(1) Six 600 ml dissolution test beakers with stirrers placed in a thermostatically controlled bath at 37°.
(2) Technicon Sample Acquisition System for Dissolution Rate Analysis (SASDRA) consisting of a peristaltic valve, a programmer and a proportioning pump III with manifold.
(3) Technicon AutoAnalyzer II system consisting of an analytical cartridge, a colorimeter, and a one-channel strip chart recorder.

A complete diagram of the system is shown in Figure 1. The main function of the system are sampling, waiting in between the samplings, and analysis of the samples. At the sampling mode of the system, for instance, the peristaltic valve opens tubes 12–19 while tubes 4–11 and 20 are closed. Each sample stream is air-segmented and each sampling probe has a glass wool filter in the bottom. When the sampling cycle is terminated the air which is forced through the D 1 connectors will keep the sampling probes clear. A detailed description of all activities of the system is given in Table I. Forward or reverse indexing of the peristaltic valve is administered from the programmer but may also be performed manually.

**Table 1 Tape program and corresponding activities of the system**

| Roller number (index) | Roller time, min. | Total time, min. | Activity of the system |
|---|---|---|---|
| 1 | 58.5 | 58.5 | Wash to testing |
| 2 | 1.5 | 60.0 | First sampling |
| 3 | 58.5 | 118.5 | Introduction of Standard 1 |
| 2 | 1.5 | 120.0 | Second sampling |
| 3 | 118.5 | 238.5 | Introduction of Standard 1 |
| 4 | 1.5 | 240.0 | Third sampling |
| 5 | 12.0 | 252.0 | Emptying of Coil 1 |
| 6 | 12.0 | 264.0 | Emptying of Coil 2 |
| 7 | 12.0 | 276.0 | Emptying of Coil 3 |
| 8 | 12.0 | 288.0 | Emptying of Coil 4 |
| 9 | 12.0 | 300.0 | Emptying of Coil 5 |
| 10 | 12.0 | 312.0 | Emptying of Coil 6 |
| 11 | 5.0 | 317.0 | Introduction of Standard 2 |
| 12 | 5.0 | 322.0 | Wash to testing |

The programmer drives a tape at a constant rate and, by punching holes in the tape, indexing is achieved at times which are determined by the distances in between the holes. An entire sampling cycle consists of three aliquots when the system is designed as described in Table I. The volume of the storage coils is 14 ml and the volume of each of the three aliquots is 3.6 ml. The third sampling is finished after 4 hours.

At this time emptying of the coils starts, one by one. The

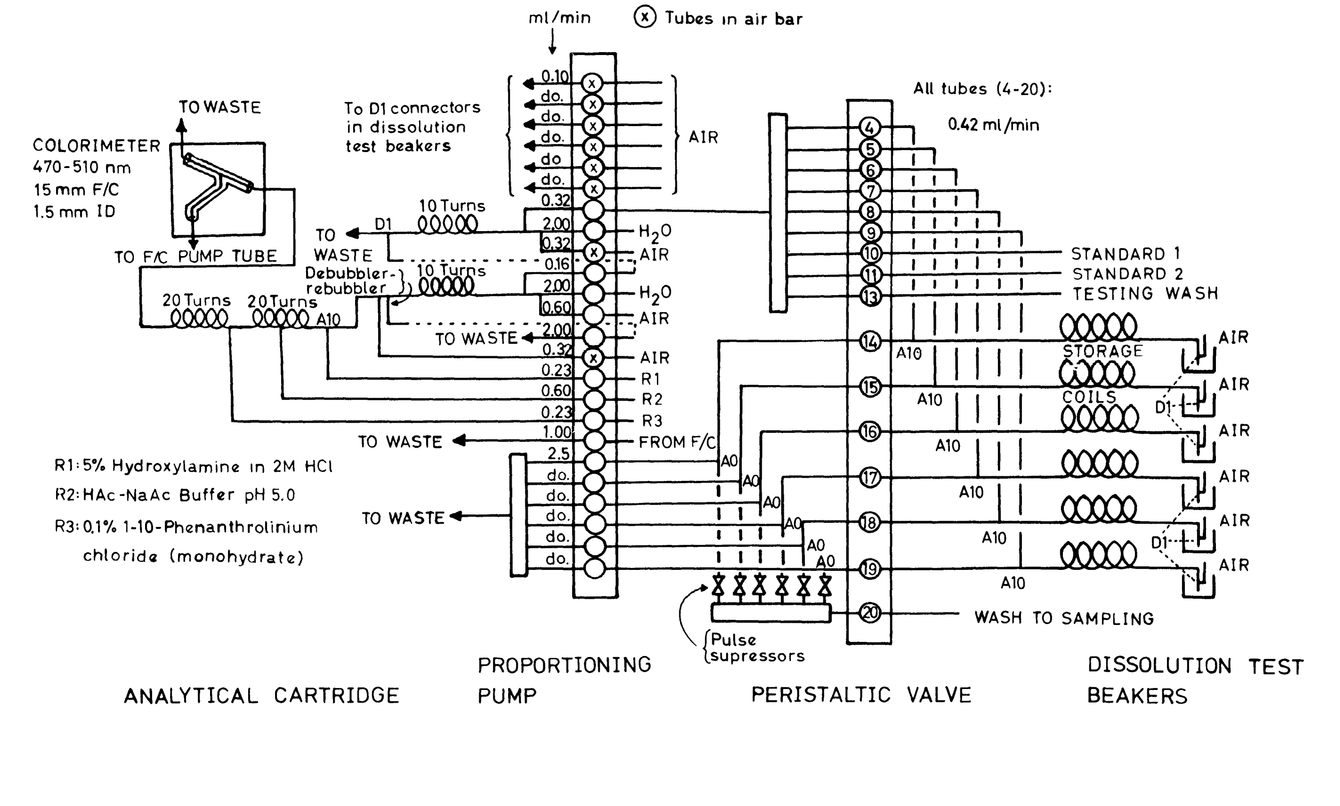

*Figure 1.*

**Table 2 Dissolution rate analysis of iron in two vitamin preparations**

| Batch | Time, h | Manual method, % specification | Automated method, % specification |
|---|---|---|---|
| | 1 | 31 | 31 |
| 145a | 2 | 51 | 50 |
| | 4 | 80 | 79 |
| | 1 | 31 | 33 |
| 146a | 2 | 52 | 53 |
| | 4 | 83 | 79 |
| | 1 | 33 | 34 |
| 147a | 2 | 53 | 54 |
| | 4 | 78 | 78 |
| | 1 | 31 | 35 |
| 148a | 2 | 52 | 54 |
| | 4 | 76 | 79 |
| | 1 | 38 | 35 |
| 149a | 2 | 57 | 56 |
| | 4 | 80 | 81 |
| | 1 | 23 | 21 |
| 81b | 2 | 41 | 40 |
| | 4 | 69 | 67 |
| | 1 | 18 | 21 |
| 82b | 2 | 36 | 39 |
| | 4 | 62 | 63 |
| | 1 | 18 | 23 |
| 83b | 2 | 36 | 40 |
| | 4 | 65 | 68 |

*a Sorbifer Durules ®*

*b Duroferon Vitamin Duretter ®*

sample is diluted in the first stage and the main stream goes to waste (Figure 1). The other stream is diluted further and split by a debubbler-rebubbler arrangement. After mixing with the reagents the absorbance is measured at a wavelength within the range 470-510 nm. When the roller indexes from 12 to 1 the system is automatically switched off. After a run it is advisable to rinse all coils and tubes with water.

### Procedure

Five tablets from each batch are placed on a stainless steel net 1.5 cm above the bottom of the dissolution beaker. A maximum of six batches can be analysed at a time (six beakers). All beakers are placed in a thermostatically controlled bath at 37°C after addition of 500 ml water (at the same temperature) to each beaker. Zero time refers to this addition of water. Stirring with steel blades (45 x 15 mm) at a rate of 40 rpm is arranged in each beaker. After about 3.5 hours the three standard solutions are introduced as Standard 1, one by one. The peaks are adjusted so a direct reading on the chart paper of the percentages is made possible. After 4 hours the third sampling starts and this is immediately followed by the emptying of the coils (see Table 1). The 50% standard is then introduced as Standard 2 to check eventual drift.

The calibration curve was found to be linear within the range 5–100% of the tablet specification (100 mg/tablet). Results of DRA are thus obtained by direct reading on the chart paper.

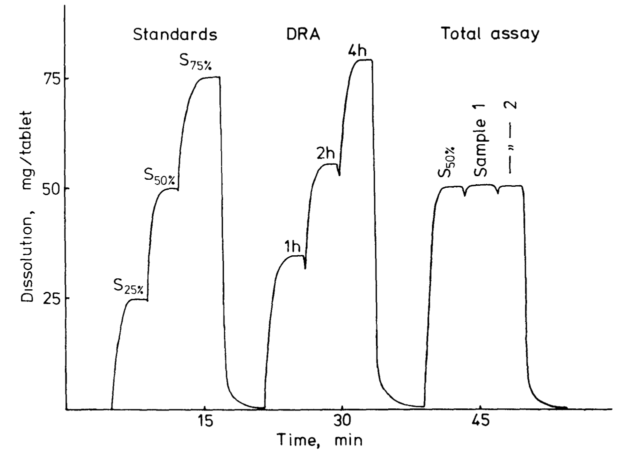

*Figure 2.*

### Results and discussion

Curves from a typical run are given in Figure 2. Readings should be made at steady state of the peaks. In Table 2 a comparison has been made between results obtained by the automated system and results obtained manually. The manual method comprises a final determination of iron by means of atomic absorption spectroscopy. The agreement is fair. The manual method includes many steps where errors might be introduced so the comparison must be made with reservation. Furthermore, the variation in the dissolution properties between individual tablets must be taken into account.

The automated system has been found to be fairly reliable and useful for quality control purposes. However, there are a few details that might be improved. The programming of the system is made by punching holes in a tape. The tape is fragile and cracks may cause interruptions or false indexing. The tubes in the peristaltic valve should not be squeezed when the system is not operational since they are rapidly flattened down so that transport of liquid is made impossible. To prevent this three screws have to be loosened each time the system is intended to be in the stand-by mode for a long period and this is inconvenient.

**REFERENCES**

[1] Shah, A.C., Peot, C.B., & Ochs, J.F. *J. Pharm. Sci.*, 1973, **62**, 671–677.
[2] Engdahl, A., Karlberg, B. & Thelander, S. *J. Pharm. Sci.*, 1976, 65, 349–352.

